# Type-2 Cytokines Promote the Secretion of the Eosinophil–Attractant CCL26 by Intestinal Epithelial Cells in Food-Sensitized Patients

**DOI:** 10.3389/fimmu.2022.909896

**Published:** 2022-06-21

**Authors:** Julián Vaccaro, Karina Eva Canziani, Luciana Guzmán, Viviana Bernedo, Marcela García, Eugenia Margarita Altamirano, Emanuel Feregotti, Renata Curciarello, Cecilia Isabel Muglia, Guillermo Horacio Docena

**Affiliations:** ^1^ Instituto de Estudios Inmunológicos y Fisiopatológicos (IIFP), UNLP, CONICET, Asociado a CIC PBA, Facultad de Ciencias Exactas, Departamento de Ciencias Biológicas, La Plata, Argentina; ^2^ Servicio de Gastroenterología, Hospital de Niños Sor María Ludovica, La Plata, Argentina; ^3^ Sala de Alergia, Hospital de Niños Sor María Ludovica, La Plata, Argentina; ^4^ Servicio de Anatomía Patológica, Hospital de Niños Sor María Ludovica, La Plata, Argentina

**Keywords:** eosinophils, CCL26, intestinal inflammation, polyps, IL-13, type-2 cytokines

## Abstract

Several inflammatory processes of the bowel are characterized by an accumulation of eosinophils at inflammation sites. The mechanisms that govern mucosal infiltration with eosinophils are not fully understood. In this work, we studied the colorectal polyp-confined tissue containing eosinophils and we hypothesized that intestinal epithelial cells are the cell source of eotaxin-3 or CCL26, a potent chemoattractant for eosinophils. We analyzed colorectal polyps (n=50) from pediatric patients with rectal bleeding by H&E staining and eosin staining, and different pro-inflammatory cytokines were assessed by RT-qPCR and ELISA. IgE and CCL26 were investigated by RT-qPCR, ELISA and confocal microscopy. Finally, the intracellular signaling pathway that mediates the CCL26 production was analyzed using a kinase array and immunoblotting in human intestinal Caco-2 cell line. We found a dense cell agglomeration within the polyps, with a significantly higher frequency of eosinophils than in control adjacent tissue. IL-4 and IL-13 were significantly up-regulated in polyps and CCL26 was elevated in the epithelial compartment. Experiments with Caco-2 cells showed that the type-2 cytokine IL-13 increased STAT3 and STAT6 phosphorylation and eotaxin-3 secretion. The addition of the blocking antibody Dupilumab or the inhibitor Ruxolitinib to the cytokine-stimulated Caco-2 cells diminished the CCL26 secretion to basal levels in a dose-dependent manner. In conclusion, our findings demonstrate a high frequency of eosinophils, and elevated levels of type-2 cytokines and eotaxin-3 in the inflammatory stroma of colorectal polyps from pediatric patients. Polyp epithelial cells showed to be the main cell source of CCL26, and IL-13 was the main trigger of this chemokine through the activation of the STAT3/STAT6/JAK1-2 pathway. We suggest that the epithelial compartment actively participates in the recruitment of eosinophils to the colonic polyp-confined inflammatory environment.

## Introduction

Human eosinophils exert substantial contributions to tissue homeostasis (1), immunity against parasites (2) and the pathogenesis of allergic inflammatory diseases (3). Eosinophils are scattered through the gastrointestinal tract, mainly in the lamina propria and to a lesser extent in the epithelial compartment. However, a high frequency of eosinophils is a hallmark of some infections and allergic inflammation. Upon activation, eosinophils secrete pre-formed granule-stored components and *de novo* synthesized molecules that promote or sustain inflammation in a wide variety of processes.

Eosinophil-associated gastrointestinal disorders are related to an abnormal immune response to certain foods, causing digestion problems. Among them, food allergies are clinical diseases mediated by hypersensitivity reactions, which, upon frequent exposure to the allergen, progress to an inflammatory condition in the gut that may involve IgE and eosinophils. It is known that most cases of tissue eosinophilia do not have a concomitant underlying IgE-mediated mechanism. In this sense, researchers are interested in studying the pathogenesis of these conditions to better understand how the immune system responds to foods and to find therapeutic alternatives.

Eosinophils reside in several mucosal tissues and the intestine is the tissue with the most significant number of tissue-resident eosinophils, both in the small and large bowel. They are mainly attracted by CCL11 or eotaxin-1 and they have a half-life of 6-7 days in the lamina propria (4). This reflects that the tissue microenvironment should be very active in promoting its homing from bone marrow and blood and in tissue survival. IL-5 is a canonical eosinophil-specific factor, which plays a central role in cell activation and survival, and along with CCL11, contributes, in homeostasis, to eosinophil recruitment into tissues (5). Other cytokines and chemokines which can be part of the type-2 inflammatory response can be involved in cell recruitment, activation and tissue remodeling. In previous studies, we reported that the colonic intestinal tissue in colorectal polyps from food-sensitized patients presented a cell infiltration dominated by eosinophils and behaved as an inductive organized tertiary lymphoid tissue. Herein, we aimed to characterize the contribution of intestinal epithelial cells in the recruitment of eosinophils through the secretion of CCL26 or eotaxin-3 in an inflammatory intestinal condition. The C-C chemokine CCL26, secreted by humans but not rodents, was also described in other mucosal tissue in an allergic context. Baumann et al. reported that patients with allergic rhinitis showed elevated levels of type-2 cytokines and CCL26, among other cytokines and chemokines in nasal lavages during the pollination season (6). Also, Provost et al. described that CCL26 exerts a more profound eosinophil-chemo attractive effect than other chemokines promoting eosinophil homing to the lung in asthmatic patients (7). To date the analysis of intestinal tissue from food-sensitized or allergic patients has seldom been performed and our work pioneered the description of the local mechanisms of IgE synthesis that contribute to the allergic process (8). The mechanisms by which CCL26 is more efficient as an eosinophil chemoattractant in an allergic context is not yet defined and here, we contribute with evidence to understand the CCL26 secretion by epithelial cells in an intestinal allergic microenvironment.

## Materials and Methods

### Patients and Tests

Patients included in this study were assisted at the Gastroenterology Unit of the Children ´s Hospital Sor María Ludovica of La Plata (Buenos Aires, Argentina). We selected patients with rectal bleeding, male (65.4%) and female, with a mean age of 5.5 years old (IQR=3.7-8.0 yo). A colonoscopy for intestinal surveillance was indicated in 104 patients and we found polyps in 48.1% (50), that were removed as part of the clinical procedure. Juvenile polyps were mainly found in the rectum (39) or sigmoid colon (11). Polyp tissues (PT) were analyzed and one biopsy from the surrounding colonic tissue (SCT) was also obtained, which was considered as a matched sample control. As additional controls, samples from adults with adenomatous (AP) (n=10) or hyperplastic colonic polyps (HP) (n=10) were analyzed.

Patients who presented polyps were also evaluated at the Allergy Unit. Allergy sensitization or food allergy was studied using clinical criteria and complementary tests. The diagnosis of food allergy is largely clinical as the double-blind placebo-controlled food challenge (DBPCFC) is not routinely performed. On suspicious, a transitory restriction diet of two weeks was carried out and food allergy or food sensitization was confirmed according to anamnesis, skin prick test (SPT), and serum IgE tests. A thorough history of dermatitis, rhinitis, asthma, and allergic responses to drugs or foods, examination and family history of atopy were considered. Skin test for food allergens was carried out in all patients with commercial extracts of cow´s milk, fish, egg, peanut, soy, and wheat; histamine phosphate (10mg/ml) and saline solution were used as controls. A skin reaction with a flare over 4 mm diameter was considered positive. Serum total and food-specific IgE (peanut, soy, and cow’s milk) were assessed in all patients by ELISA, according to Docena et al. (9).

The Ethics Committee of the Children´s Hospital of La Plata approved the protocols (Buenos Aires, Argentina) (#389-2014 and #389-2018), and patients or their parents gave written informed consent.

### Histological Analysis

Polyp and biopsy tissues were characterized by haematoxylin and eosin (H&E) or eosin staining, or with fluorochrome-conjugated specific antibodies and then analyzed by optical and confocal microscopy, respectively (8). Briefly, tissues were fixed overnight at 4°C in 3% paraformaldehyde and then embedded in paraffin as described in Mercer et al. (10). Intestinal tissue sections of 5μm were deparaffinized and stained with H&E (Biopur, Santa Fe, Argentina) or treated with sodium citrate pH 6.0 at 95°C for 15 min for antigen retrieval for immunofluorescence analysis. After blocking with 2% bovine serum albumin, incubation with FITC-conjugated anti-human IgE (diluted 1:25) (Southern Biotech, Birmingham, AL, USA) or anti-human CCL26 (diluted 1:20) (R&D Systems, Minnesota, USA) along with goat anti-IgG AlexaFluor^®^488 (diluted 1:300) (Abcam, San Francisco, USA) as a secondary antibody and propidium iodide (Sigma-Aldrich, MO, USA) was carried out. An SP5 Leica confocal microscope was used, and images were analyzed with LAS AF Lite software (Leica). Eosinophil differential staining was performed in 5μm formalin-fixed and paraffin-embedded colon tissue sections and stained with the Papanicolaou technique following the manufacturer’s protocols (Biopak, Buenos Aires, Argentina). Briefly, intestinal tissue sections were deparaffinized and stained subsequently with haematoxylin, Orange G and eosin. Sections were then dehydrated and mounted with Canadian balsam. For the eosinophil count, images were obtained in a Leica DM500 microscope with a ICC50HD camera. Cell counts were done in high-power field (HPF), which represents a 400X or 400-fold magnification, and corresponds to an area of 0.1 mm^2^ (field number of 18 mm). The presence and frequency of eosinophils was calculated according to the count of cells with *bi*-lobed *nucleus* together with their eosin-containing granules staining.

### Cytokine and IgE Levels in the Colonic Tissue

Proteins were extracted from snap-frozen PT and SCT samples by rapid rotor-stator homogenization in PBS with a protease inhibitor cocktail (SIGMA-Aldrich, MO, USA). Cytokines were quantified in the supernatants using commercial ELISA kits according to the manufacturer’s instructions: IL-4 (Immunotools, Friesoythe, Germany), IL-5 (ThermoFisher Scientific, MA, USA), IL-13 (DuoSet, R&D Systems, Minneapolis, MN, USA) and IFN-γ (DuoSet, R&D Systems, Minneapolis, MN, USA).

Total and cow’s milk-specific tissue IgE were assessed by ELISA as described in Docena et al. (9). Briefly, polystyrene microtiter wells (NUNC, Maxisorp, Denmark) were coated with anti-human IgE polyclonal antiserum (100µl, 400µg/ml) (Sigma, MO, USA) for total IgE quantification or allergens (cow’s milk, soy or peanut proteins-10ug/ml) for specific IgE. Wells were blocked with 5% equine serum in saline phosphate buffer. Tissue lysates were added and incubated for 1h at 37°C, followed by incubation with biotinylated goat anti-human IgE (ϵ-chain specific) antibody (Vector Laboratories, Cat# BA-3040, RRID : AB_2336144) (1/2000, 1h at 37°C) followed by streptavidin-horseradish peroxidase (Sigma, MO, USA) (1/10,000, 1h at 37°C) for total IgE quantification, or with anti-human IgE conjugated to alkaline phosphatase (Sigma, MO, USA) (1:1000, 2hs at 37°C) for specific IgE. For quantification, a calibration curve was prepared in duplicate using a reference standard (1000 IU/ml) for total IgE. As substrate, *ortho*-phenylenediamine (MP Biomedicals, CA, USA) and peroxide were used for total IgE, the reaction was stopped with 2M H_2_SO_4_ and optical density (OD) was measured at 492 nm. For specific IgE, *ρ*-nitrophenyl phosphate (Sigma, MO, USA) was used, the reaction was stopped with 0,1N EDTA and OD was measured at 405 nm.

### Cell Culture

Caco-2 cells were grown in DMEM supplemented with 10% FBS, 100 U/ml penicillin, 100 mg/ml streptomycin and 110 mg/L pyruvate. Confluent Caco-2 cells grown on 24-well plates were fasted for 12h and then stimulated with recombinant human (rh)-IFN-γ (10ng/ml), rh-IL-4 (100 ng/ml) or rh-IL-13 (10ng/ml) (R&D Systems, Minneapolis, MN, USA) for 90 min, for further immunoblotting assays. Chemokines in cell-culture supernatants were assayed after stimulation with FliC (1µg/ml), rh-IFN-γ (10ng/ml), rh-IL-4 (100 ng/ml), rh-IL-13 (10ng/ml), rh-IL-5 (10ng/ml) (R&D Systems, Minneapolis, MN, USA), Dupilumab (human anti-interleukin-4 receptor alpha monoclonal antibody) (50µg/ml) (Sanofi Genzyme, Regeneron, NY, USA) or Mepolizumab (human anti-interleukin-5 monoclonal antibody) (50 µg/ml) (GSK, San Polo di Torrile, Italy) for 48h at 37°C. We also used a specific JAK1 and JAK2 inhibitor, Ruxolitinib (Novartis, Stein, Switzerland), at different doses for 2h. The inhibitors and monoclonal antibodies were diluted in medium and incubated with cells alone or in combination with the different stimuli.

### Gene Expression Analysis

Polyp and biopsy samples were received in RNA Later Solution (Biogenex) and stored at -80°C. Total RNA was isolated using the Illustra RNAspin Mini RNA Isolation Kit (GE Healthcare, Little Chalfont, UK) following the manufacturer’s protocols. The isolated RNA was then reverse transcribed using random primers and M-MLV Reverse transcriptase (Invitrogen, CA, USA). Real-time quantitative PCR (qRT-PCR) for CCL5, CCL26, IFN-γ, IL-4, IL-5 and IL-13 was performed using SYBR Green PCR Master Mix (BioRad, CA, USA) and the iCycler thermal cycler (Bio-Rad, CA, USA). Transcript expression levels were normalized to β-actin (whole tissue) or RPLP0 (epithelial compartment) as house-keeping genes. Comparative threshold (CT) method was used for data analysis. Data were expressed as relative quantitation of gene expression (2^−ΔCt^).

Primer sequences used are listed in **Table 1** and the running protocol was the same for all primers.

**Table 1:** Primer sequences employed in the gene expression analysis.

Gene	Forward	Reverse
IFN-γ	AATTGGAAAGAGGAGAGTGAC	CATTCATGTCTTCCTTGATGG
IL-13	CAGTTCAACTGAAACTTCG	TCTGCAACTTCAATAGTCAG
IL-4	CGACTGCACAGCAGTTCCA	AGGTTCCTGTCGAGCCGTTT
IL-5	CATCGAACTCTGCTGATAGCC	CATCGAACTCTGCTGATAGCC
CCL-5	CCCATATTCCTCGGACAC	TCTTTCGGGTGACAAAGC
CCL-26	ACACGTGGGAGTGACATATCCA	GACTTTCTTGCCTCTTTTGGTAGTG
β-ACTIN	CCTGGCACCCAGCACAAT	GCCGATCCACACGGAGTACT
RPLP-0	GCAATGTTGCCAGTGTCTG	GCCTTGACCTTTTCAGCAA

### Immunoblotting

Cells were lysed and harvested with 10 mM HEPES, 1.5 mM MgCl2, 10 mM KCl and 0.1% Igepal in the presence of a protease inhibitor mixture (Sigma-Aldrich) and sodium vanadate (VO_4_Na) as a phosphatase inhibitor. Total protein amount was determined with the bicinchoninic acid assay (Pierce, Rockford, IL, USA). Protein extracts from Caco-2 cells were resolved on 10% SDS-PAGE gels under reducing conditions (BioRad Mini-Protean III; BioRad, Richmond, CA, USA), and then transferred onto a nitrocellulose membrane (Bio-Rad Laboratories, Hemel Hempsted, UK) for 1h at 300 mA. Blots were blocked and probed with a mouse anti-STAT6 and anti-phospho STAT6, diluted 1:600 and 1:2000 respectively (Santa Cruz, Santa Cruz, CA, USA) or with a mouse anti-STAT3 and anti-phospho STAT3 diluted 1:600 and 1:300 respectively (Santa Cruz, Santa Cruz, CA, USA) as primary antibodies. HRP-conjugated goat anti-mouse IgG diluted 1:3000 was used as a secondary antibody (BioRad, Hercules, CA, United States). β-actin was used as a loading control using a rabbit anti-β-actin primary antibody diluted 1:2000 (Abcam, Cambridge, MA, United States). HRP-conjugated goat anti-rabbit IgG diluted 1:3000 was used as a secondary antibody (BioRad, Hercules, CA, United States). Protein bands were visualized by enhanced chemiluminescence reagents (ECL Plus; GE Healthcare, Danderyd, Sweden). The bands were scanned with C digit scanner (LI-COR Biosciences, Lincoln, Nebraska, United States) and quantified using ImageJ software.

### ELISA Assay for CCL26

CCL26 was quantified by ELISA in stimulated Caco-2 cell supernatants and cell lysates from colonic tissues, following the manufacturer’s instructions (DuoSet, R&D Systems, Minneapolis, MN, USA).

### Kinase Signaling Pathway Assay

Confluent Caco-2 cells were fasted for 12h and then stimulated with rh-IFN-γ (10ng/ml) or rh-IL-13 (10ng/ml) (R&D Systems) for 5, 10, 45 and 90min. Cells were lysed and harvested as previously described in the presence of the protease inhibitor mixture and phosphatase inhibitor. The protein content of lysates was adjusted to 600µg/ml. The coated nitrocellulose membranes (PathScan RTK Signaling Antibody Array Kit, Cell Signaling Technology) were incubated with cell lysates over night at 4°C. The kinases captured by the antibody-coated spots were detected with a cocktail of biotinylated specific antibodies, then HRP-conjugated streptavidin and finally it was developed with Enhanced chemiluminescence reagents (ECL Plus; GE Healthcare, Danderyd, Sweden), following the manufacturer’s instructions. Membranes were scanned with the C digit scanner. The images were analyzed with the Image-J software.

### Statistical Analysis

Comparison between paired groups was made using *Student´s t*-test. Results were expressed as mean ± standard error of the mean (SEM) or violin plots. Differences between means were considered statistically significant when p<0.1. GraphPad Prism 8.00 (San Diego, CA, USA) was used for the statistical analysis.

## Results

### The Stroma of Colorectal Polyps Showed an Eosinophil-Dense Inflammatory Cell Infiltration and Type-2 Cytokine-Dominated Environment

**Figure 1:**
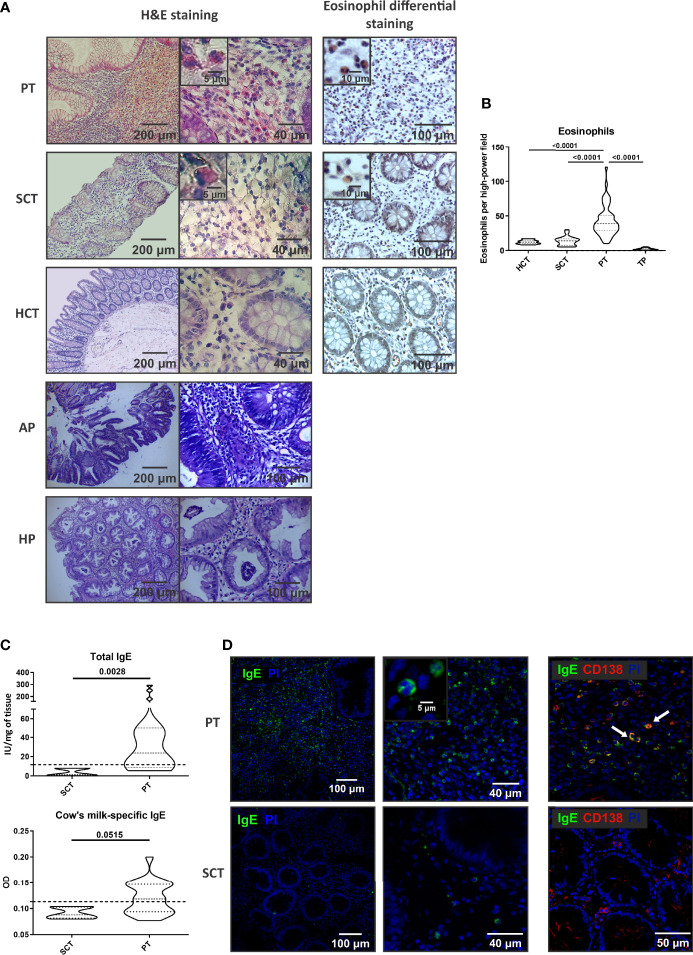
Cell infiltrate and IgE in the stroma of colorectal polyps. **(A)** Histology by H&E and eosin staining of tissue sections from polyp tissues (PT), surrounding control tissue (SCT) and heathy control tissue (HCT). Adenomatous polyps (AP) and hyperplastic polyps (HP) stained with H&E are also depicted. Representative images are depicted. **(B)** Eosinophil count in colonic tissues from pediatric patients and from adults with tumoral polyps (TP). **(C)** Total and milk-specific IgE quantification in the tissues. **(D)** IgE and CD138 staining by confocal microscopy in the tissues (double- staining are marked with arrows and it corresponds to IgE-expressing plasma cells). Data are expressed as the mean value ± SEM. Statistic differences were calculated using *Student´s t*-test.

We have previously reported that colonic polyps found in children with rectal bleeding and abdominal pain are mostly single, protruded to the lumen of the bowel, and are mainly located in rectum (48). The histological analysis of the stroma showed a prominent cell infiltration in the lamina propria of PT, whereas the paired SCT and colonic tissues of healthy subjects (HCT) showed a normal tissue cellularity. Eosinophils were visualized with the eosin-based staining and we found a significant higher frequency of eosinophils in PT than in SCT or HCT (43,06+/-22,32 vs 13,75+/-6,67 and 12,10+/-2,98 eosinophils/HPF, respectively). Adenomatous and hyperplastic colonic polyps from adults were also analyzed, and we found a significantly reduced eosinophil count (**Figures 1A, B**). Tumoral or malignant polyps are infrequent in the pediatric population.

To further elucidate the mechanisms that promoted the recruitment of eosinophils to the polyp, we analyzed the inflammatory environment. Tissue IgE antibodies were elevated in PT compared to SCT (p<0.005), with significantly higher levels of milk-specific IgE in PT than in SCT (**Figure 1C**). Furthermore, we found that IgE^+^ cells were scattered throughout all the polyp tissue and the co-staining with anti-CD138 antibody revealed the presence of plasma cells (IgE^+^CD138^+^ cells) and eosinophils (IgE^+^CD138^-^ cells with a bi-lobed nucleus) (**Figure 1D**). Previous works demonstrated that serum IgE correlated with polyp tissue IgE (8). Here, we found that patients with polyps showed high levels of total serum IgE (90%) and food specific IgE (74-88%), and personal or family history of atopy or allergy (**Table 2**).

**Table 2:** Demographics of pediatric patients with rectal bleeding and juvenile polyps.

Characteristic	Subjects (n=50)
**Age (y), median (IQR)**	5.50 (3.70 – 8.0)
**Total IgE level (IU/mL), median (IQR)**	212.10 (67.65 – 444.53)
**Total IgE level > 60 UI/mL, n (%)**	45 (90)
**Cow’s milk protein (CMP)-specific IgE, n (%)**	40 (80)
**Peanut-specific IgE, n (%)**	44 (88)
**Soy-specific IgE, n (%)**	37 (74)
**SPT, n (%)**	1 (2)
**Food-specific IgE, n (%)**	42 (92.3)
**Atopic condition (personal or family), n (%)**	30 (60)
**Dermatitis**	8 (16)
**Urticaria in response to CMP**	4 (8)
**Asthma**	4 (8)

**Figure 2:**
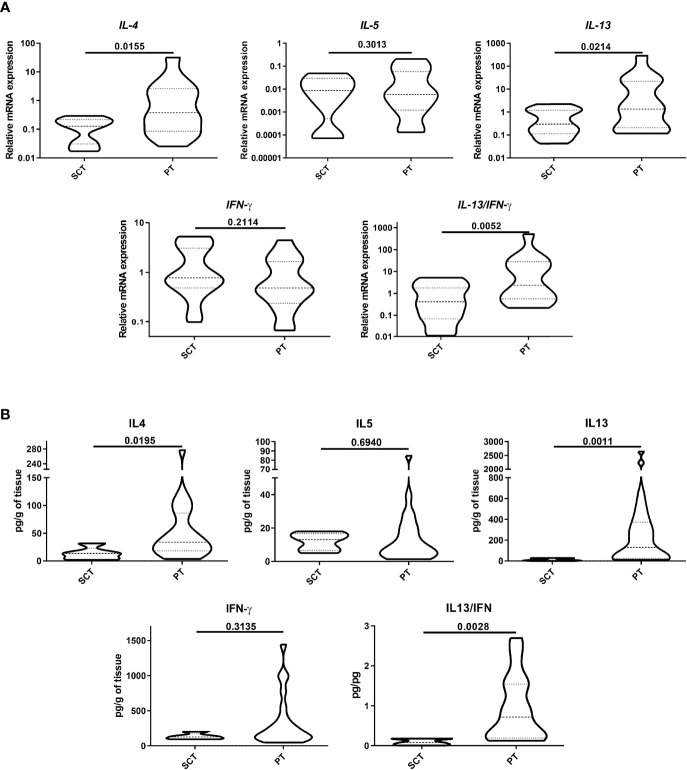
Analysis of cytokine production. Protein expression **(A)** and quantification **(B)** of type-1 and -2 cytokines were analyzed by RT-qPCR and ELISA in tissue lysates. Data are expressed as the mean value ± SEM. Statistic differences were calculated using *Student´s t*-test.

We next analyzed the production of type-1 and type-2 cytokines in the colonic tissues, comparing the cytokine expression and protein levels in PT and SCT. As shown in **Figure 2A**, IL-4 and IL-13 transcripts in PT were significantly higher than in SCT, whereas IFN-γ remained unchanged. IL-5 showed an increasing trend in PT compared to SCT. The IL-13/IFN-γ ratio was higher in PT than in SCT (p<0.01). Also, we found significant higher protein levels of IL-4 and IL-13 than in SCT, whereas IFN-γ remained statistically unchanged, while the IL-13/IFN-γ ratio was increased in PT compared to SCT (p<0.01) (**Figure 2B**). These findings indicate that the tissue microenvironment within the polyps is dominated by type-2 cytokines, which could be produced by ILC-2, eosinophils and Th2 cells.

### CCL26 Was Differentially Produced in the Epithelial Compartment of Polyps

**Figure 3:**
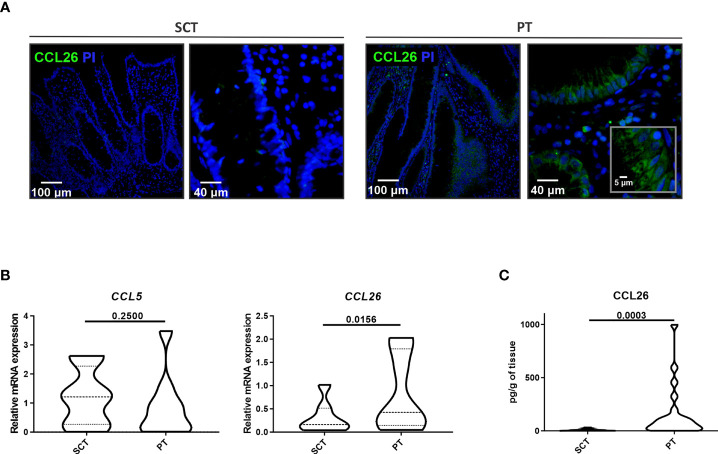
Analysis of CCL26 production in colonic tissues. **(A)** CCL26 production was analyzed by confocal microscopy (DAPI was used for nucleus staining). The epithelial compartment showing CCL26 cytosolic expression is magnified. **(B)** CCL5 and CCL26 expression measured by RT-qPCR was analyzed in colonic tissues. **(C)** The CCL26 tissue level was quantified by ELISA. Data are expressed as the mean value ± SEM. Statistic differences were calculated using *Student´s t*-test.

Given that CCL26 is a potent chemoattractant for eosinophils, we next analyzed the presence of this chemokine in the colonic tissue of food-sensitized patients. We found by confocal microscopy an increased expression of CCL26 in the epithelial compartment of PT whereas it was undetectable in SCT (**Figure 3A**). As shown in the magnified picture, CCL26 staining was observed in the cytosol of epithelial cells. CCL5 (RANTES) and CCL26 (eotaxin-3) transcript quantification showed a significantly higher expression of CCL26 in PT than SCT, whereas CCL5 remained unchanged (**Figure 3B**). Moreover, the quantification of CCL26 in tissue lysates showed a higher concentration in PT than in SCT (p<0.01) (**Figure 3C**).

### IL-13 Induced the Secretion of CCL26 Through the Activation of the STAT/JAK Pathway in Colonic Epithelial Cells

**Figure 4:**
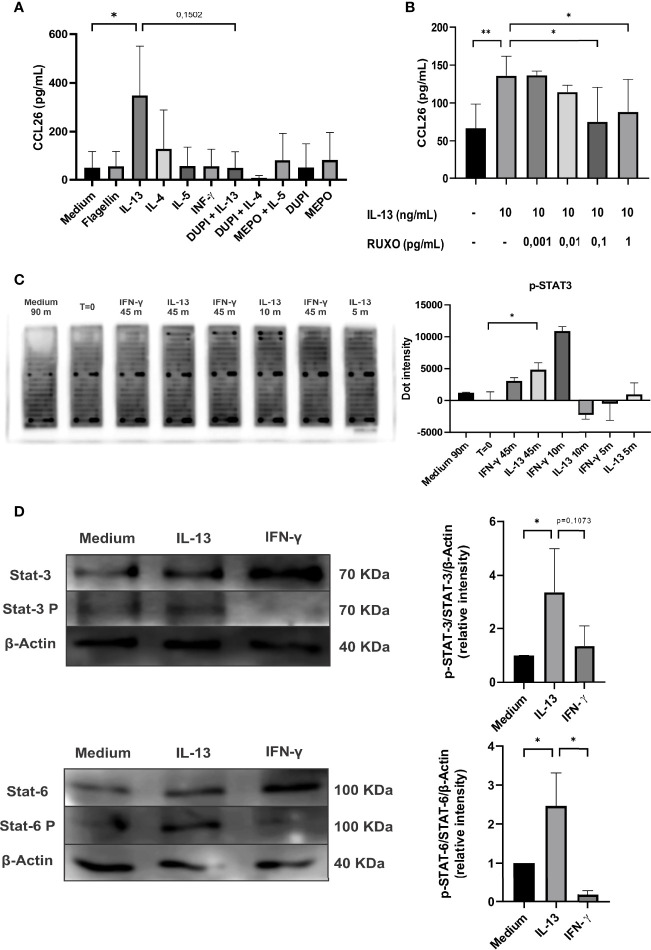
Analysis of CCL26 production by Caco-2 cells in response to type-1 and -2 cytokines. **(A)** Chemokine quantification was measured in the culture supernatants by ELISA after 48h of cytokine stimulation. Blocking antibodies were used (DUPI: Dupilumab or anti IL-4αR monoclonal antibody; MEPO: Mepolizumab or anti-IL-5 monoclonal antibody). **(B)** IL-13-induced CCL26 secretion was inhibited with RUXO (Ruxolitinib or JAK1/2 inhibitor). **(C)** Image of array slides of Caco-2 cell lysates after IL-13 (10 ng/ml) or IFN-γ (10 ng/ml) stimulation at different time-points (0, 5, 10, 45 minutes). Quantification of the array signal for phosphorylated STAT3 is shown. **(D)** Immunoblot analysis for STAT3 and STAT6 activation on lysates from Caco-2 cells stimulated with IL-13 (10 ng/ml) or IFN-γ (10 ng/ml) for 90min. Semi-quantitative analysis of the results are shown as pSTAT-6/STAT-6/β-actin and pSTAT-3/STAT-3/β-actin signal intensity ratios. Each column represents mean ± standard error (*P< 0,1, **P< 0,01).

These findings prompted us to study the role of type-2 cytokines in the production of CCL26 by human colonic epithelial cells. We found in Caco-2 cells that IL-13, but not IL-5, differentially promoted the secretion of CCL26 (p<0.05). Medium, flagellin (Caco-2 cells express TLR-5) and IFN-γ were included as controls and none of them induced the chemokine secretion. We found no significant induction of CCL26 expression upon IL-4 stimulation. To confirm that CCL26 secretion was specifically induced by IL-13, we blocked its receptor with Dupilumab (an IL-4Rα chain specific monoclonal antibody) *in vitro*. We also suppressed the IL-4 stimulation with Dupilumab and IL-5 stimulation with Mepolizumab (an anti-IL-5 monoclonal antibody). We found that Dupilumab abrogated the IL-13-induction of CCL26 secretion, indicating that the IL-13R is involved in the signaling pathway leading to CCL26 production (**Figure 4A**). As the IL-13R is linked to the intracellular STAT/JAK kinase, we co-incubated Caco-2 cells with IL-13 and Ruxolitinib, a JAK1 and JAK2 inhibitor. We observed an inhibition of the IL-13-induced CCL26 secretion in a dose-response manner, thus suggesting that the STAT/JAK pathway is involved in eotaxin-3 production (**Figure 4B**).

Finally, to identify the specific intracellular pathways promoted by the extracellular IL-13 stimulus that provokes the CCL26 secretion, we carried out a phosphokinase array. We found that STAT3 was phosphorylated when cells were incubated with IL-13 or IFN-γ (**Figure 4C**). In addition, the literature shows that IL-13 also induces STAT6 phosphorylation (11). Therefore, we analyzed by immunoblotting STAT3, p-STAT3, STAT6 and p-STAT6 in Caco-2 cell lysates. **Figure 4D** shows that IL-13 induced p-STAT3 and p-STAT6 and increased the ratio p-STAT3/STAT3 and p-STAT6/STAT6 (p<0.1). Overall, these findings indicate that IL-13 promoted the phosphorylation of STAT3 and STAT6 through the IL-13 receptor in the colonic epithelial cell line, and that the activation of the JAK 1/2 promoters induced the transcription and secretion of CCL26.

## Discussion

The gut-associated lymphoid system includes organized lymphoid tissue such as mesenteric lymph nodes, Peyer’s patches, and lymphoid follicles within the lamina propria. This system comes into direct contact with food antigens, which may explain why as many as 50% of food allergic disorders present with gastrointestinal manifestations. Even though 2% of ingested food antigens are absorbed, tolerance develops through a variety of mechanisms (e.g., T-cell anergy or induction of regulatory T cells) that protect against allergy development. In a sensitized host, a food antigen can bind to IgE, activating and releasing several potent mediators and cytokines leading to mucosal inflammation. Several inflammatory processes of the bowel are characterized by an accumulation of eosinophils at sites of inflammation. The mechanisms that govern mucosal infiltration with eosinophils are not fully understood. In this study, we hypothesized that the colonic epithelial cells release eotaxin-3 and we demonstrated that the type 2 cytokine IL-13 increased eotaxin-3 mRNA levels and eotaxin-3 protein secretion in the Caco-2 human intestinal epithelial cell line. We previously reported that the allergic inflammation detected in juvenile polyps of patients with food-sensitization was accompanied with IgE synthesis (8). Furthermore, the presence of food-specific IgE exclusively within the polyp, and not in the surrounding mucosal tissue, indicates that IgE is locally produced and probably induced or enhanced by the presence of food allergens that gain access to tissue from the intestinal lumen. We studied the inflammatory environment of the confined stroma within colonic polyps and we observed a dense cell infiltration with a high frequency of eosinophil. We here propose that intestinal epithelial cells that line the mucosa are key triggers of the eosinophil recruitment through the secretion of eotaxin-3 or CCL26 upon stimulation with IL-13.

As in the small intestine, colonic eosinophils are considered to represent normal constituents of the mucosa when singly dispersed in the lamina propria (9-26 eosinophils/HPF). Eosinophils are more numerous in the intestinal lamina propria of the healthy proximal colon of pediatric subjects, with 20–50 eosinophils per HPF and infiltrating the crypt epithelium (12). In our study, we found that the control or surrounding colonic tissue of the distal colon harbors 13-20 eosinophils/HPF, whereas the polyp tissue showed a significantly higher frequency of cells (43,06+/-22,32 eosinophils/HPF), along with significantly increased levels of CCL26 in the epithelial compartment.

Other authors have reported similar results in nasal polyps, with a high tissue content of chemokines and high eosinophil count (13). Furthermore, CCL26 has also been described as produced by human bronchial epithelial cells (14) and keratinocytes (15) and its imbalanced secretion, probably due to an overexpression of type-2 cytokines, was implicated in atopic dermatitis and asthma (16–18). Nevertheless, CCL26 has not been described in food allergy. The overexpression of IL-13 has also been described as promoting mucosal barrier disruption and further amplifying inflammation with tissue remodeling (19–22). In our study, we detected IL-13 at higher levels than IL-4 and IL-5 in the stroma of polyps and this prompted us to analyze the effect of this cytokine on human colonic epithelial cells.

Regarding the human gut, it has been reported in different cell lines that CCL26 expression is induced by IL-13 and IL-4 through the STAT6 pathway (11). Lan et al. reported that intestinal specimens from patients with colorectal cancer showed up-regulated expression of CCL26 in advanced tumor stages (23), which is associated with colorectal cancer malignancy. In addition, STAT-6 regulates CCL26 expression in esophageal cells (24) and it has been strongly associated with inflammation in esophageal biopsies from patients with eosinophilic esophagitis (25). In contrast, there was no significant correlation between CCL5 and inflammation, considering that eosinophils express its cognate receptor CCR1.

Blanchard et al. demonstrated that the human colonic epithelial cell lines HT-29 CL.19A and T84 secreted eotaxin-3 upon stimulation with IL-4 or IL-13 in a dose-dependent manner (11). In our work, we found a significant induction of CCL26 secretion in Caco-2 cells upon stimulation with IL-13, which was reversed after blocking its cognate receptor and with a JAK inhibitor, thus demonstrating that this type-2 cytokine promoted the activation of the two canonical STAT/JAK intracellular pathways, STAT 3 and STAT 6, to activate the eotaxin-3 promoter.

In conclusion, our results demonstrate that the stroma of polyps from patients with food-sensitization contain a dense cell infiltration dominated by eosinophils and type-2 cytokines. Epithelial cells of polyps, unlike epithelial cells from the normal adjacent mucosa, produced the main eosinophil-chemoattractant eotaxin-3. Importantly, we provide evidence that STAT 3 and STAT 6 are necessary for the IL-13-driven CCL26 gene up-regulation and secretion in Caco-2 cells. Overall, these findings suggest that the allergic inflammatory content of polyps may promote the peripheral eosinophil recruitment at the site of inflammation and that the inhibition of JAK 1/2 may control this process.

## Data Availability Statement

The original contributions presented in the study are included in the article/supplementary material. Further inquiries can be directed to the corresponding author.

## Ethics Statement

The studies involving human participants were reviewed and approved by The Ethics Committee of the Children´s Hospital of La Plata approved the protocols (Buenos Aires, Argentina) (#389-2014 and #389-2018). Written informed consent to participate in this study was provided by the participants’ legal guardian/next of kin.

## Author Contributions

All authors have made substantial contributions to the study: -Conception and design of the study: RC, CIM and GD. -Acquisition of data: JV, KC, LG, VB, MG, EA EF. -Removal of the polyp: LG and VB. -Analysis and/or interpretation of data: JV, KC, LG VB, MG, EA, EF, RC, CIM, GD. -Drafting the manuscript: GD. -Revising the manuscript critically for relevant intellectual content: JV, KC, LG, VB, MG, EA, EF, RC, CIM, GD. -Approval of the version of the manuscript to be published: JV, KC, LG, VB, MG, EA, EF, RC, CM, GD.

## Funding

This work was supported by grants from Agencia Nacional de Promoción Científica y Tecnológica (PICT 2018-2479) and UNLP (grant 11/X695) to GD.

## Conflict of Interest

The authors declare that the research was conducted in the absence of any commercial or financial relationships that could be construed as a potential conflict of interest.

## Publisher’s Note

All claims expressed in this article are solely those of the authors and do not necessarily represent those of their affiliated organizations, or those of the publisher, the editors and the reviewers. Any product that may be evaluated in this article, or claim that may be made by its manufacturer, is not guaranteed or endorsed by the publisher.
